# AI-driven high-risk pregnancy prediction: balancing early detection, anxiety, and discrimination in digital public health

**DOI:** 10.3389/fpubh.2026.1752484

**Published:** 2026-03-26

**Authors:** Qingqing Ji, Mengyi Wang

**Affiliations:** 1 West China Second University Hospital, Sichuan University, Chengdu, Sichuan, China; 2 Key Laboratory of Birth Defects and Related Diseases of Women and Children (Sichuan University), Ministry of Education, Chengdu, Sichuan, China

**Keywords:** algorithmic bias, artificial intelligence, data privacy, health equity, high-risk pregnancy, patient-centered risk communication, perinatal risk prediction, predictive anxiety

## Abstract

Over the past five years, perinatal risk prediction using artificial intelligence has expanded rapidly, drawing on routine clinical records, ultrasound findings, and continuous physiologic signals to generate dynamic high-risk scores across pregnancy. These tools promise earlier identification of complications, more precise monitoring, and better targeting of preventive resources, but their net benefit will hinge on how risk labels shape care and lived experience. In this Perspective, we conducted a targeted, non-systematic narrative synthesis integrating (i) evidence on AI-based obstetric risk prediction, (ii) lessons from prenatal screening and high-risk labeling, and (iii) principles and guidance on trustworthy digital health, equity/fairness, risk communication, and reproductive-data governance to examine how probabilistic outputs can unintentionally increase distress and inequity. We argue that risk labeling may fuel predictive anxiety when probabilities are interpreted deterministically, and secondary anxiety when intensified surveillance is experienced as confirmation of danger. We also outline discrimination pathways, including biased data and labels that over-flag socially disadvantaged groups, defensive clinical escalation that drives over-medicalization, and social or employment harms when sensitive pregnancy data are reused beyond care. To balance benefit and harm, we propose integrated safeguards: transparent model documentation, local and subgroup calibration, continuous fairness monitoring, structured and patient-centered risk communication with meaningful choice, strict privacy and purpose-limitation protections, and tiered psychological support embedded in clinical pathways. Future deployments should proceed as monitored pilots that jointly track clinical outcomes, equity, and perinatal mental health before scale-up.

## 1 Introduction

Over the past five years, perinatal risk prediction has emerged as a flagship artificial intelligence (AI) use case in digital public health. Large-scale electronic health records (EHRs), obstetric ultrasound and other imaging, and increasingly continuous physiologic and wearable signals are now being integrated in multimodal machine-learning pipelines, enabling dynamic risk scoring across pregnancy and the peripartum period worldwide (1). Recent systems illustrate this shift—for example, early preeclampsia prediction using population-level data and bedside risk engines (2, 3), and postpartum hemorrhage models trained on longitudinal EHR trajectories (4). This rapid growth is often framed as an accuracy race, but accuracy-first narratives can overlook a parallel question: what happens to pregnant people after an algorithm labels them “high-risk”?

**Figure 1:**
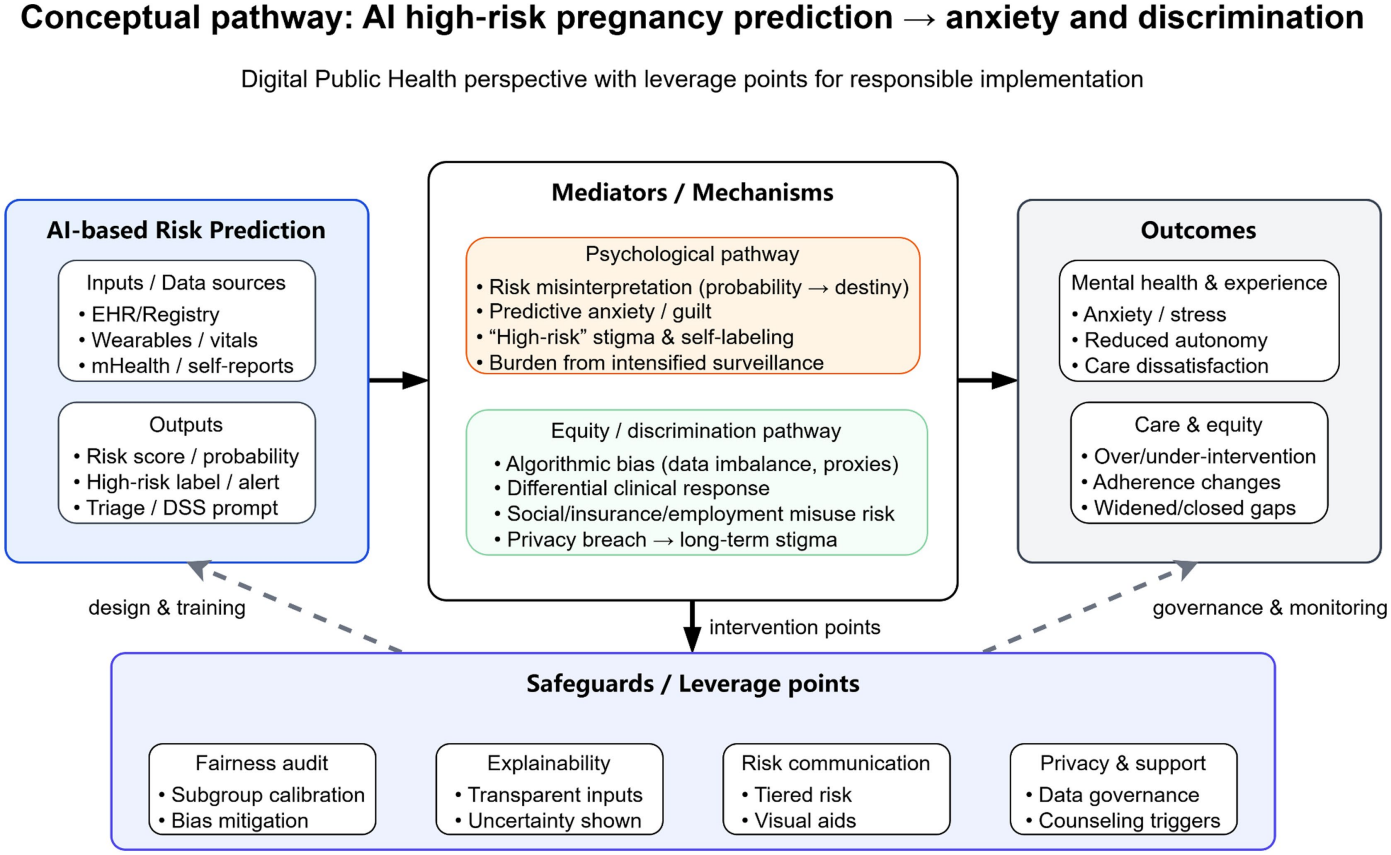
Conceptual pathway: AI high-risk pregnancy prediction → anxiety and discrimination. The figure is organized from left to right. The left panel shows the AI-based risk-prediction stage, including typical data inputs (e.g., EHR/registry data, wearable or vital-sign data, and mHealth/self-reports) and outputs (e.g., risk scores, high-risk labels/alerts, and triage or decision-support prompts). The central panel presents two mediating pathways. The psychological pathway summarizes how probabilistic risk information may be misinterpreted as deterministic, contributing to predictive anxiety, guilt, self-labeling, and burden from intensified surveillance. The equity/discrimination pathway summarizes how algorithmic bias, differential clinical response, privacy breach, and possible social misuse of risk status may translate prediction into stigma, discrimination, or widened inequities. The right panel groups downstream consequences into two broad outcome domains: mental health and care experience (e.g., anxiety/stress, reduced autonomy, dissatisfaction) and care/equity impacts (e.g., over- or under-intervention, adherence changes, and widened or narrowed gaps). The bottom panel shows cross-cutting safeguard or leverage points—including fairness audit, explainability, risk communication, and privacy/support—that can act at multiple stages of the pathway through design, training, intervention, governance, and monitoring. The figure is conceptual and intended to clarify hypothesized mechanisms and implementation targets rather than to depict a fully established causal chain. AI, artificial intelligence; EHR, electronic health record; mHealth, mobile health; DSS, decision support system.

If implemented well, these tools could shift care from reactive rescue to earlier, targeted prevention and more precise monitoring, while improving triage and informing regional resource allocation. That potential is highly relevant to public health: preventing major obstetric complications is central to lowering maternal mortality and advancing Sustainable Development Goal target 3.1, which calls for a global maternal mortality ratio below 70 per 100,000 live births by 2030 (5). Yet AI-based “high-risk” labels may also heighten anxiety, stigma, or self-blame; foster misinterpretation and over-medicalization; and amplify social discrimination or inequity. This Perspective evaluates whether AI high-risk labeling can inadvertently intensify anxiety and discrimination and proposes a balancing framework for digital public health deployment. Figure 1 maps the conceptual pathway and key safeguard points linking AI risk prediction to these outcomes.

To improve transparency, Section 1.1 summarizes the purpose, scope, and evidence synthesis approach for this Perspective.

### 1.1 Methods and evidence synthesis

This article is a Perspective grounded in a targeted, non-systematic narrative synthesis rather than a formal systematic review. The literature cited in this Perspective is intended to be illustrative rather than exhaustive and is used to support conceptual development rather than to provide a comprehensive review of the field. Accordingly, we selected representative examples from obstetric AI prediction, prenatal screening and high-risk labeling, and digital health governance to clarify mechanisms, implementation challenges, and safeguard options relevant to this argument. We integrated three complementary evidence streams: (i) obstetric AI risk-prediction research (including exemplar models, validation studies, and reviews of prediction performance and deployment contexts); (ii) lessons from prenatal screening and “high-risk” labeling, together with perinatal mental health evidence on how probabilistic risk information can shape anxiety, distress, and care-seeking; and (iii) principles and guidance on trustworthy digital health, including fairness and equity monitoring, risk communication and shared decision-making, and governance of sensitive reproductive data. Evidence was identified through targeted searches and citation-chaining from key reviews, consensus guidance, and representative empirical studies, prioritizing work from approximately the past five years while drawing on seminal conceptual frameworks where necessary. We used a mechanism-first mapping approach to connect AI outputs and clinical workflows to downstream psychological and equity harms (Sections 2–6), and then mapped each challenge to actionable safeguards and measurable implementation outcomes proposed for monitored pilots prior to scale-up (Section 7). No new participant data were collected for this Perspective.

## 2 Clinical and public health value of AI-based prediction

The value of perinatal AI prediction lies not in producing a risk score per se, but in whether it improves timely, actionable decisions for individuals and supports equitable allocation of preventive capacity across populations. In practice, AI adds the most when it (1) detects risk earlier or more continuously than conventional tools, (2) stratifies who benefits from what intensity of care, and (3) ties predictions to specific, evidence-based actions embedded in clinical and public-health workflows (1).

### 2.1 Clinical benefits

By learning from multimodal signals—EHR histories, laboratory trends, ultrasound or other imaging, and continuous physiologic streams such as wearables or intrapartum cardiotocography—AI models can surface risk trajectories earlier than traditional single-time-point scores and update them as new data arrive (1). This shifts prediction from a static label to a dynamic decision aid: earlier stratification helps match monitoring intensity to need, while iterative updating supports escalation or de-escalation as risk evolves. In preeclampsia, the Pre-eclampsia Integrated Estimate of Risk machine-learning model more clearly distinguishes pregnant people at very low versus very high short-term risk of severe outcomes, improving triage, transfer decisions, and intensive care unit preparedness compared with clinical judgment alone (3). Across other high-burden scenarios (e.g., gestational diabetes mellitus, postpartum hemorrhage, preterm birth, fetal compromise), models built on routine antenatal or intrapartum data can identify elevated risk before standard screening windows or clinical deterioration, enabling earlier counseling, prophylaxis, and safer routing to higher-acuity settings when indicated (6–10). The clinical promise is less about “automation” than about tighter, timelier care loops—prediction → targeted prevention → rapid escalation—that reduce missed opportunities and delay-driven harm.

These gains depend on robust local calibration and patient-centered risk communication linked to clear action pathways, rather than standalone ‘high-risk’ labels.

### 2.2 Public health impact

At scale, the same risk engines can be embedded in regional EHR platforms or mobile-health programs to support population screening and longitudinal case management (1, 11, 12). Risk-stratified registries allow systems to prioritize scarce resources—transport, referral slots, high-dependency beds, aspirin prophylaxis for high preeclampsia risk, or diabetes care programs—toward those most likely to benefit, including in low-resource EHR settings where prevention must be targeted to be effective (13–15). When predictions are tracked over time, they can also feed dashboards that show where risk clusters, where referral pathways fail, and whether interventions are narrowing or widening gaps across geography or socioeconomic groups—supporting Sustainable Development Goal-aligned maternal-mortality reduction strategies (5).

Public-health value is sustainable only with explicit equity safeguards and strong data-governance and privacy protections; otherwise, tools may reproduce structural inequities and cause downstream psychological or social harms.

## 3 Psychological impact: does AI increase pregnancy-related anxiety?

### 3.1 High-risk labeling and “predictive anxiety”

“Predictive anxiety” refers to distress that arises when a pregnant person receives a probabilistic AI risk score but experiences it as a verdict. Numeric outputs or binary labels (“high-risk”) can invite deterministic framing—probability is heard as inevitability—especially when counseling is brief or the system is presented as uniquely objective. The emotional response may be immediate: shock, fear, a sense of lost control, and anticipatory worry (16–18).

Prenatal screening communication offers a close analogue. Systematic evidence shows that false-positive or “high-risk/suspected” screening results significantly heighten short-term anxiety even when later ruled out (16). Qualitative studies of suspected fetal-anomaly screening describe intense worry driven by uncertainty and clinicians’ inability to provide immediate clarity (17). With Non-Invasive Prenatal Testing, unexpected or ambiguous findings commonly provoke distress during the waiting period, illustrating how uncertain risk information can amplify catastrophic interpretation (18).

AI prediction is not identical to a one-off test: scores may be updated throughout pregnancy and can reshape care pathways. Still, the psychological ingredients overlap—probabilistic uncertainty, waiting for outcomes, and a strong “authority signal” that nudges people to treat risk as certainty. AI labeling may therefore reproduce these anxiety-prone dynamics earlier in gestation and at scale, and may intensify them through repeated re-notifications. This inference is mechanism-based rather than directly demonstrated in obstetric AI deployment studies and should therefore be read as a conceptually grounded extrapolation from adjacent evidence.

### 3.2 Secondary anxiety from intensified medical surveillance

AI-flagged risk rarely arrives alone; it typically triggers stepped-up surveillance—additional labs, more clinic visits, serial ultrasounds, home blood-pressure or glucose monitoring, and intensified fetal testing such as cardiotocography. For some women this is reassuring, but for others it generates “secondary anxiety.” Frequent medical encounters can tacitly confirm the belief that “something is wrong,” reinforcing fear of fetal harm, guilt about one’s body, and vigilance fatigue. Studies of women labeled high-risk for spontaneous preterm birth show elevated anxiety, underscoring how risk status and intensive follow-up can coexist with psychological burden (19).

A brief vignette makes the mechanism concrete: if an AI dashboard shifts a woman from “moderate” to “high” preeclampsia risk at 22 weeks, she may be scheduled for weekly visits, home blood-pressure uploads, and an additional ultrasound. Even when clinicians intend reassurance, the sudden escalation can feel like implicit confirmation of danger (“my body is failing”), worsening sleep and heightening hypervigilance between visits. The point is not that surveillance is inherently harmful, but that its psychological meaning depends on how the rationale, uncertainty, and available support are communicated.

### 3.3 Research gaps

Despite rapid deployment, there is almost no direct empirical work on how AI obstetric risk prediction affects perinatal mental health. Most studies prioritize accuracy and calibration, while reviews note that patient-centered psychological outcomes are rarely measured in digital maternity innovations (20). This omission matters because antenatal anxiety is common early in pregnancy and socially patterned (21), and prenatal distress has measurable downstream effects on mothers and offspring (22). At present, the evidentiary base is therefore uneven and should be interpreted accordingly. Some elements of the pathway are supported indirectly by adjacent empirical literatures: prenatal screening and high-risk labeling studies support the claim that uncertain or false-positive risk information can increase short-term anxiety, while evidence from intensified monitoring contexts suggests that more frequent surveillance can coexist with psychological burden. By contrast, there is still very little direct evidence on how pregnant patients specifically interpret or respond to AI-generated obstetric risk labels in routine care. For this reason, several links in Figure 1 should be read as conceptually plausible and mechanism-informed rather than as fully established causal effects of AI deployment itself. These pathways are also likely to vary across health systems and cultural contexts, because the meaning of a “high-risk” label is shaped by counseling practices, baseline trust in clinicians and technology, medico-legal pressures, access to follow-up care, insurance and employment protections, and local stigma surrounding pregnancy complications or mental distress.

Future research should use longitudinal, mixed-methods designs to track anxiety, depression, traumatic stress, and care-seeking changes after AI labeling, with an explicit equity lens. A feasible starting point is to embed validated Patient-Reported Outcomes (e.g., Edinburgh Postnatal Depression Scale–Anxiety subscale, Generalized Anxiety Disorder-7 scale, State–Trait Anxiety Inventory-short form) into prospective roll-outs using stepped-wedge or interrupted time-series designs, paired with qualitative sampling of how women interpret and act on risk outputs (23–25). Evidence from fetal-abnormality care suggests that timely counseling and psychological supports can mitigate distress (26)—an implementation lesson AI programs should test rather than assume.

To this point, we have focused on the individual-level psychological pathways through which AI-labeled risk can generate predictive and secondary anxiety. However, in digital public health these effects are co-produced with structural features of the system—who is labeled, how labels travel, and what consequences follow. We therefore treat privacy protection, bias control, and stigmatization risks not as separate “technical” topics, but as boundary conditions that can amplify anxiety and translate prediction into discrimination, motivating the governance safeguards in Section 6.

## 4 Structural pathways to anxiety and discrimination: bias, stigmatization, and privacy misuse

Section 4 adds the sociotechnical boundary conditions that determine whether these psychological pathways are amplified, unequally distributed, or translated into discrimination in real deployments. We distinguish upstream technical determinants—who is more likely to be labeled high-risk and how widely that label can travel (bias and privacy)—from downstream social enactment—how labels reshape clinical interaction and can generate stigmatization or discrimination. Accordingly, Section 4 is organized as a layered pathway from upstream determinants (Sections 4.1–4.2) to downstream enactment (Sections 4.3–4.4). Across these layers, the common mechanism is higher perceived stakes with reduced controllability: when a label is more frequent, more visible, and more consequential, uncertainty is more readily experienced as threat, amplifying anxiety and undermining trust. This framing also clarifies why Section 6 consolidates the safeguards: recommendations must jointly address communication practices and structural risks, rather than alternating problem–solution statements across Sections 4–5.

### 4.1 Algorithmic bias and inequity (model/data layer)

Bias in obstetric AI often starts upstream. Training datasets can over-represent people who are wealthier, urban, insured, and consistently engaged with care, while under-representing minority ethnic groups, migrants, rural families, or those with multimorbidity and fragmented access. In EHR-based models, patterns of care use (missed visits, late booking, fewer labs) may function as proxies for social disadvantage, so algorithms end up labeling “structural exposure to scarcity” as “biological risk.” Consensus guidance warns that this kind of label bias—systematic over- or under-labeling of specific groups—can scale inequity unless datasets are transparently described, audited, and performance is reported by subgroup (27–29). In practice, inequitable labeling can also amplify anxiety by making “high-risk” status feel both more common and less contestable in already disadvantaged groups; we translate mitigation steps into implementable safeguards in Section 6 (27–31). Recent calls for intersectionality-aware and transdisciplinary AI governance further reinforce that fairness cannot be reduced to aggregate performance alone; it must also account for how overlapping social positions shape labeling burden, interpretability, and downstream harm in deployment.

### 4.2 Privacy risks and secondary use (data infrastructure/ecosystem layer)

Pregnancy and reproductive data are uniquely sensitive and long-tailed: leakage can generate persistent stigma, legal vulnerability, or discrimination long after birth. Female-oriented Technologies apps and wearables collect granular cycle, sexual, and pregnancy-intention data, and investigations show that some commercial platforms share or monetize these datasets for profiling and targeted advertising, with documented harms when data are repurposed for employment or insurance decisions (32, 33). Privacy, fairness, and trust are coupled: weak privacy protections magnify inequity and undermine willingness to engage with digital maternity care. World Health Organization AI governance therefore frames privacy and equity as co-requirements for trustworthy deployment (34, 35). We operationalize privacy-by-design and accountability measures in Section 6.

### 4.3 Clinical interaction and medical stigmatization (clinical interaction layer)

Once a pregnancy is algorithmically tagged high-risk, clinical thresholds may drift downward in some settings. “High-risk” outputs can heighten clinicians’ perceived uncertainty and encourage defensive practice—more tests, earlier referrals, and sometimes a lower threshold for induction or cesarean “just in case,” even when evidence for incremental benefit is thin (36, 37). Labels also shape expectations and communication. Clinicians may (often unintentionally) adopt a more paternalistic tone, narrowing shared decision-making around surveillance intensity or mode of birth. For patients, this can feel like being managed as a risk object rather than a partner, eroding autonomy and trust. Importantly, this is an implementation risk to be tested empirically, not an inevitable outcome. Good Machine Learning Practice therefore emphasizes that AI should support—not replace—clinical judgment and that users must understand uncertainty and intended use to avoid automated escalation (31). We consolidate the corresponding workflow and communication safeguards in Section 6.

### 4.4 Downstream social discrimination (societal layer)

Risk scores do not necessarily stay in the clinic. In contemporary data ecosystems, “high-risk probability” could be sought, purchased, or inferred by employers, insurers, data brokers, or even family members through third-party sharing of wearables or Female-oriented Technologies app data, workplace health-monitoring programs, or insurance risk-stratification models that incorporate reproductive or physiologic signals (34, 38). In cultural and household contexts where fertility is moralized, a high-risk label can intensify blame or control (“you caused this risk”), compounding anxiety and discouraging care-seeking—why ethical reviews in obstetrics flag downstream discrimination as a real-world hazard (34, 37). These societal consequences are analytically downstream, but they feedback psychologically by raising perceived stakes and making the label harder to ignore or renegotiate; Section 6 specifies governance safeguards to limit such spillovers.

## 5 Risk communication and informed consent challenges

### 5.1 Misinterpretation of probabilistic AI outputs

AI risk tools typically return probabilistic estimates, yet pregnant people and families may interpret these outputs more deterministically—especially when absolute risk, baseline comparators, and uncertainty are not explicitly communicated (39–42). Evidence from machine-learning-supported clinical encounters shows that both clinicians and patients struggle to translate scores into personally meaningful information, and many users defer to model outputs because they appear mathematically precise or system-endorsed (41, 42). This “AI authority illusion” can intensify emotional shock, narrow perceived choices, and encourage conformity even when clinical context supports alternative interpretations (43, 44). Downstream, such deference may fuel unnecessary compliance or over-medicalization—accepting intensified surveillance, earlier referral, or intervention “just in case,” rather than a proportionate plan grounded in shared decision-making.

### 5.2 Need for structured risk communication models

Trustworthy-AI guidance stresses that predictions must be embedded in communication pathways rather than delivered as raw numbers (35, 45, 46). In real-world maternity care, probabilistic predictions are often interpreted under time pressure and heightened stakes; without explicit context (baseline risk, uncertainty, and model limits), “risk” can be experienced as a verdict rather than fallible evidence, intensifying predictive and secondary anxiety. Risk-communication research shows that how probabilities are framed materially affects understanding and perceived threat, particularly among patients with lower numeracy (39, 40, 47, 48). Section 6 therefore consolidates the implementable safeguards that translate model outputs into shared decision-making workflows while making uncertainty and limits explicit.

### 5.3 Consent and autonomy

A core ethical question is whether pregnant people can refuse AI prediction or algorithmic labeling. If risk scoring is default with no opt-out, consent becomes procedural rather than meaningful. The World Health Organization guidance and the FUTURE-AI framework (Fairness, Universality, Traceability, Usability, Robustness, Explainability, Accountability, Inclusiveness) explicitly link trustworthy deployment to autonomy, transparency, and non-coercive use of sensitive reproductive data (35, 45, 46). At minimum, programs should (1) offer a genuine opt-out when clinically feasible, (2) explain in plain language—supported by simple visuals—what the score means, its uncertainty, and its limits, and (3) document intended use (what decisions it may inform and what it cannot decide), with an explicit commitment that clinical judgment and patient values remain central (43, 44). High-risk notification pathways should also integrate psychological support and referral so that risk information does not inadvertently become harm.

## 6 Recommendations for responsible, equitable, and anxiety-sensitive AI

Table 1 summarizes the core benefits, unintended psychological and equity harms, and essential safeguards across the AI prediction pipeline. Importantly, we treat anxiety-mediated harms and structural discrimination risks (Section 4) as coupled in practice: trust, labeling, and downstream consequences connect these levels. Table 1 therefore serves as a practical bridge to the recommendations below.

Detailed, disease-specific matrices (including preeclampsia, gestational diabetes mellitus, postpartum hemorrhage, preterm birth, and obstetric intensive care unit triage) are provided in Supplementary Tables S1–S5.

**Table 1:** Core benefits, risks, and safeguards for AI-based high-risk pregnancy prediction.

Core domain	Key benefits	Key risks (anxiety/discrimination)	Essential safeguards
Model development	Early risk stratification at scale	Data imbalance → biased high-risk labeling	Multi-site training; subgroup fairness audit
Validation/calibration	Real-world accuracy for prevention/triage	Poor calibration → false alarms & anxiety	External validation; local calibration; error bounds
Clinical deployment (EHR/DSS)	Timely escalation & resource allocation	Alert fatigue; defensive over-intervention	Clinician-in-the-loop; clear thresholds; audit over-triage
Risk communication	Personalized counseling & prevention plans	Probability misread as destiny → distress	Tiered + absolute risk; visual aids; “probability ≠ destiny” script
Follow-up/monitoring	Earlier detection of deterioration	Surveillance burden; widened access gaps	Tele-follow-up options; equity-stratified monitoring
Data governance	System-level surveillance & QI	Privacy breach; social/insurance misuse	Strict access control; ban non-medical secondary use
Psychological support	Improves coping and adherence	High-risk label triggers guilt/panic	Counseling triggers; routine anxiety screening

This condensed matrix summarizes cross-cutting domains relevant to obstetric AI prediction tools, highlighting key public-health benefits, potential psychological (anxiety-related) and equity (discrimination-related) harms, and practice-ready safeguards. AI, artificial intelligence; EHR, electronic health record; DSS, clinical decision support system; SES, socioeconomic status; QI, quality improvement.

### 6.1 Transparency and explainability

To reduce “AI authority illusion” and deterministic framing (Sections 3.1 and 5.1), models should include model cards and dataset documentation that clearly state purpose, intended populations, inputs, development setting, and known limitations (27, 45). Explainability should be communication-ready: display uncertainty, justify thresholds, and specify human–AI teaming rules so outputs are treated as decision support, not destiny (45).

### 6.2 Fairness auditing and bias mitigation

To prevent algorithmic and label bias (Section 4.1), models need multi-population calibration and stratified performance reporting by socioeconomic status, ethnicity/immigration status, rurality, age, and comorbidity patterns before deployment and continuously thereafter (28, 45, 49). Audits should recur as case mix and workflows change (e.g., the Prediction Model Risk of Bias Assessment Tool for AI), and teams should identify and constrain harmful proxies (such as visit frequency or insurance type) so access scarcity is not misread as biology (27, 28, 49, 50). This also argues for an intersectionality-aware approach to fairness governance, in which explainability, subgroup performance, and the social meaning of model outputs are reviewed together by transdisciplinary teams rather than by technical metrics alone (51). In practice, fairness monitoring should be prespecified at deployment and repeated at regular review intervals, with more frequent review during early rollout and less frequent review once performance is stable. Monitoring should include subgroup assessment of false-positive rates, false-negative rates, calibration, alert frequency, referral patterns, and follow-up completion across clinically and socially relevant strata such as age, socioeconomic position, rurality, ethnicity/immigration status, and comorbidity burden. Material disparities should trigger documented review of thresholds, input features, proxy variables, workflow effects, and communication practices, with temporary restriction of use if inequitable harm cannot be promptly mitigated.

### 6.3 Standardized AI-risk communication guidelines

To reduce predictive/secondary anxiety and deterministic interpretations of probabilistic outputs, health systems should operationalize a standardized communication bundle that treats AI as fallible evidence for shared decisions—not a verdict to obey (41, 45). Concretely: (1) translate model outputs into tiered pathways explicitly linked to options and next steps (e.g., what additional monitoring is offered, what can be deferred, and what triggers escalation) (40, 44); (2) communicate risk as absolute risk with baseline comparators, and when helpful use natural-frequency formats (e.g., “out of 100 pregnancies similar to yours…”) to make denominators and incremental risk differences explicit (39, 40); (3) use simple visuals (icon arrays/pictographs/risk ladders) to support patients with lower numeracy (47); (4) state uncertainty, intended use, and subgroup limits (including known bias risks) so outputs remain discussable rather than determinative (45, 48, 49). (5) Preserve agency to restore perceived controllability by explicitly offering “decision space” (recommended vs. optional), documenting patient preferences, and enabling reasoned overrides in EHR-integrated workflows (41–45). This last step matters because when an algorithm-endorsed pathway is perceived as the only “safe” option, declining or negotiating recommendations can feel morally risky, shifting shared decision-making toward compliance and eroding perceived autonomy (41, 43, 45). Workforce training should combine digital literacy with empathic scripts and shared decision-making skills to translate AI outputs into choices rather than commands (45, 46).

### 6.4 Privacy and protection against misuse

To block discrimination via data reuse (Sections 4.3–4.4), pregnancy risk data require stricter safeguards than routine analytics. Policies should prohibit non-medical use (employment, insurance, commercial profiling) and enforce purpose limitation, access logging, and independent audits. Technical measures should prioritize data minimization, tiered access, and transparent third-party contracts, including across Female-oriented Technologies and wearable ecosystems (34, 35, 52).

### 6.5 Integrated psychological-support approach

To keep high-risk labeling from becoming harm or stigma (Sections 3 and 4.2), notifications should trigger stepped support: immediate interpretation and reassurance, brief follow-ups, and mental-health referral if distress persists. Embedding short anxiety/depression/trauma screens in AI workflows and linking results to obstetric and mental-health services aligns with calls from the American College of Obstetricians and Gynecologists and the World Health Organization for repeated perinatal screening and routine maternal-mental-health integration (53, 54).

To make these safeguards implementation-ready, deployment plans should specify who monitors fairness, who governs model changes, and which workflow actions are triggered by an AI-generated high-risk alert. Fairness monitoring should be built into deployment governance through prespecified review intervals and documented escalation procedures when subgroup disparities, performance drift, or inequitable downstream consequences emerge. Decisions about model recalibration, updating, threshold changes, or temporary suspension of use should be overseen through a multidisciplinary governance structure involving obstetric clinical leadership, data science, quality and safety personnel, and, where feasible, ethics, equity, and patient-representation input. This group should document the rationale for any threshold revision, workflow modification, or restriction of use. Operationally, this could be embedded as a trigger-response workflow within routine obstetric care: an AI-generated high-risk alert prompts clinician review, confirmation that the output is clinically plausible, and use of a standardized communication script explaining the risk estimate, its uncertainty, and the rationale for any change in monitoring or referral. At the same contact, the patient undergoes a brief distress screen and a check for practical barriers to attendance, monitoring adherence, or care access. Based on predefined local criteria, the encounter is then routed to routine follow-up, enhanced surveillance, mental-health referral, or social-support referral, with completion of each step documented in the clinical record for audit and quality review.

## 7 Discussion and conclusion

### 7.1 Discussion

AI-driven risk prediction could materially advance earlier detection, better triage, and scalable prevention in maternity care. Yet its real-world value will hinge on what happens after a score enters everyday workflows—how accountability is shared, how services absorb referrals, and how risk is communicated. Throughout this discussion, we distinguish three linked levels of impact: patient-level experience (anxiety, interpretation, and trust), clinician-level response (decision-making, escalation, and communication), and system-level consequences (capacity, equity, governance, and population distribution of benefit and harm). Integrated into EHR order sets or regional dashboards, models can shift responsibility: clinicians may feel obliged to comply under liability pressures, and pregnant people may treat any departure as unsafe. In that framing, questions and preferences can become harder to voice, because deviating from an algorithm-endorsed pathway is interpreted as “accepting risk.” This reduces the practical space for shared decision-making (recommended vs. optional choices) and can shift decisions from deliberation toward compliance, thereby eroding autonomy (41, 42, 45). In rural or resource-limited settings, a high-risk label may prompt referrals beyond local service capacity, transferring time, travel, and financial costs to families. When these downstream frictions are absent from model design, prediction that is statistically “fair” may still yield inequitable care. Evaluation should therefore move beyond AUC and calibration to implementation and patient-centered outcomes—workload and referral churn, out-of-pocket spending, and patient-reported autonomy, anxiety, and trust—while risk thresholds are co-designed to match service capacity, reviewed as capacity changes, and governed transparently with community input. A responsible digital public-health approach requires integrated guardrails—fairness audits, meaningful explainability, psychological safety, ethical oversight, patient-centered risk communication, and privacy protection—treated as one system rather than optional add-ons. To make this operational, Box 1 maps each challenge to the supporting evidence base, actionable safeguards, and measurable outcomes to track in monitored pilots prior to scale-up.

BOX 1 **Challenge-to-safeguard mapping and outcomes to monitor in monitored pilots.**

Predictive anxiety (deterministic interpretation of probabilities) → Evidence base: prenatal screening/high-risk labeling; risk communication; perinatal mental health (16, 39, 54) → Safeguards: structured, patient-centered risk communication (absolute risk + comparators; uncertainty framing; teach-back; meaningful choice) → Outcomes to monitor: risk comprehension, decisional conflict, anxiety/distress symptoms, satisfaction/trust, engagement with recommended care.

Secondary anxiety (surveillance experienced as confirmation of danger) → Evidence base: intensified monitoring and illness vigilance; perinatal mental health (54–57) → Safeguards: tiered surveillance pathways with explicit rationale; minimize non-actionable alerts; proactive check-ins; embedded psychological support for those flagged at higher risk → Outcomes: perceived surveillance burden, sleep/functional impact, distress trajectories, adherence to monitoring, unscheduled contacts/visits.

Bias and over-flagging of socially disadvantaged groups → Evidence base: algorithmic bias/fairness; obstetric prediction external validity (49, 50) → Safeguards: local and subgroup calibration; threshold governance; continuous fairness monitoring; periodic model updating with drift checks → Outcomes: subgroup false-positive/false-negative patterns, downstream intervention rates by subgroup, inequities in timeliness/quality of care and outcomes.

Differential clinical response and defensive escalation (over-medicalization) → Evidence base: high-risk labeling effects; clinician/patient interaction with AI decision aids; implementation considerations (41, 44) → Safeguards: pathway-based decision support with escalation guardrails; audit-and-feedback on actions triggered by risk flags; shared decision documentation → Outcomes: escalation intensity (admissions, additional tests), intervention rates, iatrogenic harms, patient-reported experience measures, cost/resource use.

Privacy risks and misuse of sensitive pregnancy data beyond care → Evidence base: digital health governance; reproductive-data privacy; FemTech regulatory gaps (33, 35) → Safeguards: strict purpose limitation; access controls and audit logs; minimization; clear consent/notice; restrictions on secondary use and sharing → Outcomes: access/log anomalies, privacy incidents, patient-reported trust and willingness to disclose information, opt-out rates.

Downstream social harms (stigma, insurance/employment consequences, discrimination) → Evidence base: discrimination pathways; workplace discrimination risk from wearables; governance gaps and secondary-use risks (38, 52) → Safeguards: governance and policy protections limiting external reuse; support and referral pathways for affected patients; institutional accountability mechanisms → Outcomes: reported discrimination events, care avoidance, continuity of care, trust, subgroup differences in engagement and outcomes.

Adjacent evidence from digital pregnancy contexts suggests that online health-information behaviors can be associated with heightened pregnancy-related anxiety (cyberchondria), while structured home-monitoring/telemedicine models may also reduce anxiety when embedded in supportive clinical communication—highlighting that the mental-health impact of prediction-like technologies depends on how uncertainty and follow-up are designed (55–57). Going forward, interdisciplinary teams spanning obstetrics, nursing/midwifery, mental health, data science, ethics, law, public health, and patient advocates should treat each rollout as a monitored pilot. Pilots should pair subgroup calibration with perinatal-anxiety measures (e.g., Edinburgh Postnatal Depression Scale–Anxiety subscale/Generalized Anxiety Disorder-7 scale trajectories) and pre-specified stop rules before scale-up.

This point can also be situated within established implementation science and clinical AI evaluation frameworks.

This argument is also consistent with established implementation and evaluation frameworks. From an implementation science perspective, the Consolidated Framework for Implementation Research helps explain why the same predictive tool may produce different downstream effects across settings depending on workflow design, organizational readiness, leadership engagement, staffing, communication routines, and local perceptions of usefulness or burden (58). The Reach, Effectiveness, Adoption, Implementation, and Maintenance framework likewise suggests that real-world value cannot be inferred from model performance alone, because population impact also depends on who is reached, whether clinicians adopt the tool, how consistently it is implemented, and whether its use is sustained over time (59). In the clinical AI literature, early-stage evaluation frameworks such as Developmental and Exploratory Clinical Investigations of Decision Support Systems Driven by Artificial Intelligence and reporting guidance such as the Consolidated Standards of Reporting Trials–Artificial Intelligence and the Standard Protocol Items: Recommendations for Interventional Trials–Artificial Intelligence similarly emphasize the importance of human–AI interaction, integration into routine care, and context-sensitive evaluation beyond technical accuracy (60–62). Related work in maternal digital health further suggests that the effects of digital tools in pregnancy depend not only on technical functionality, but also on implementation quality, service context, and program evaluation in real care settings (63). Read together, these frameworks reinforce our central claim: deployment conditions and sociotechnical context shape whether an AI-generated high-risk label improves care, intensifies anxiety, or reproduces inequity.

### 7.2 Conclusion

AI-driven high-risk pregnancy prediction can improve earlier detection and preventive targeting, but its net benefit depends on post-score implementation—how risk labels reshape communication, surveillance intensity, referral burden, and autonomy. Deployment should therefore proceed as monitored pilots with explicit governance (fairness, privacy, and accountability guardrails) and patient-centered endpoints (anxiety, trust, decisional quality, and equity of care) tracked alongside clinical outcomes before scale-up.
