# Supplementary material for: AI-driven high-risk pregnancy prediction: balancing early detection, anxiety, and discrimination in digital public health
Source: Front Public Health. 2026 Mar 26;14:1752484. doi: 10.3389/fpubh.2026.1752484 (PMC13062171; doi:10.3389/fpubh.2026.1752484)
Supplement: Supplementary file 1 [file Table_1.DOCX]

**Table S1. PE prediction pipeline (full matrix)**

| **Domain/Stage** | **Benefits** | **Harms (anxiety/discrimination)** | **Mitigations** |
| --- | --- | --- | --- |
| **1. Data sourcing & cohort definition** | Captures early PE signals across pregnancy; enables large-scale risk stratification | Selection bias if only tertiary/high-referral cohorts; missing early ANC users may understate risk in disadvantaged groups | Multi-level data (primary→tertiary); transparent inclusion/exclusion; document missingness patterns |
| **2. Feature selection (BP, labs, Doppler, history)** | Combines multi-modal cues for earlier, more accurate prediction | Use of proxies (residence/insurance) may encode SES bias; overfitting to site-specific lab patterns | Limit proxies; clinically grounded features; fairness-aware feature review |
| **3. Model training** | Learns nonlinear trajectories (e.g., BP rise patterns) beyond rule-based tools | Imbalanced PE prevalence inflates false positives in low-risk groups | Class-imbalance handling; cross-site CV; report calibration by trimester |
| **4. External validation** | Tests generalizability for early- vs late-onset PE; reduces missed high-risk cases | Poor external performance may amplify inequities (under-detection in rural/low-SES) | Validate in varied settings; subgroup performance disclosure |
| **5. Calibration to local incidence** | Produces actionable absolute risk estimates | Miscalibration → high FP labeling → anxiety, unnecessary monitoring/admissions | Local recalibration; publish PPV/NPV + uncertainty bounds |
| **6. EHR/DSS deployment** | Real-time PE early-warning alerts; triggers prophylaxis and escalation | Alert fatigue; defensive admissions/iatrogenic preterm birth; labeling becomes identity | Clinician-in-the-loop; clear escalation thresholds; audit intervention drift (admission, MgSO₄, early delivery) |
| **7. Risk communication** | Enables personalized counseling and prevention plans | Risk interpreted as certainty; fear of stroke/eclampsia; family pressure | Tiered risk + absolute risk visuals; “probability ≠ destiny” script; document informed consent |
| **8. Follow-up intensity & surveillance** | Earlier detection of deterioration; prevents severe PE | Surveillance burden; unequal follow-up access widens PE gaps | Match intensity to absolute risk + preferences; tele-BP reviews; equity-stratified follow-up monitoring |
| **9. Post-deployment monitoring** | Maintains accuracy with changing protocols/populations | Drift increases FP/bias silently → trust erosion | Continuous recalibration; equity/harm dashboards; transparent updates/retirement |

**Abbreviations:** PE, preeclampsia; BP, blood pressure; ANC, antenatal care; SES, socioeconomic status; FP, false positive; CV, cross-validation; PPV, positive predictive value; NPV, negative predictive value; EHR, electronic health record; DSS, decision support system; MgSO₄, magnesium sulfate; tele-BP, tele–blood pressure monitoring.
