# Supplementary material for: AI-driven high-risk pregnancy prediction: balancing early detection, anxiety, and discrimination in digital public health
Source: Front Public Health. 2026 Mar 26;14:1752484. doi: 10.3389/fpubh.2026.1752484 (PMC13062171; doi:10.3389/fpubh.2026.1752484)
Supplement: Supplementary file 2 [file Table_2.DOCX]

**Table S2. GDM prediction pipeline (full matrix)**

| **Domain/Stage** | **Benefits** | **Harms (anxiety/discrimination)** | **Mitigations** |
| --- | --- | --- | --- |
| **1. Early pregnancy data capture (BMI, hx, labs)** | Identifies high-risk women before OGTT window; supports preventive lifestyle counseling | Data gaps in early ANC users (migrants/rural) create biased risk estimates | Ensure early ANC linkage; quantify missingness by subgroup |
| **2. Feature engineering (glucose trends, diet/PA, PCOS, family hx)** | Improves prediction beyond single fasting value | SES/education proxies may unfairly inflate risk for vulnerable groups | Remove/adjust SES proxies; clinically anchored features |
| **3. Training with imbalanced prevalence** | More sensitive early GDM detection | FP risk creates diet anxiety or restrictive behavior | Cost-sensitive training; report FP rate and downstream burden |
| **4. External validation** | Confirms cross-population use | Poor generalization to ethnic/BMI subgroups → discrimination | Multi-ethnic validation; subgroup AUC/calibration |
| **5. Calibration / risk thresholds** | Generates actionable absolute risk for prevention | Over-low thresholds → over-labeling → distress | Local calibration; clinically agreed thresholds |
| **6. Integration into prenatal EHR/mHealth** | Supports targeted OGTT timing, monitoring, coaching | Over-surveillance; digital burden; alert fatigue | Clinician-confirmed prompts; limit alert frequency |
| **7. Patient risk communication** | Enhances understanding of preventive behaviors | “High-risk” label triggers guilt, fear for fetus, family blame | Tiered absolute-risk framing; emphasize modifiable risk; provide supportive messaging |
| **8. Lifestyle/tele-coaching follow-up** | Improves adherence, reduces progression | Digital divide excludes low-literacy/rural groups | Offer low-tech alternatives; usability testing in vulnerable groups |
| **9. Post-deployment monitoring** | Tracks impact on GDM incidence & outcomes | Drift or coach-bias widens disparities | Equity-stratified monitoring of OGTT completion, treatment uptake, outcomes |

**Abbreviations：**GDM, gestational diabetes mellitus; OGTT, oral glucose tolerance test; BMI, body mass index; hx, history; PA, physical activity; PCOS, polycystic ovary syndrome; FP, false positive; ANC, antenatal care; SES, socioeconomic status; EHR, electronic health record; mHealth, mobile health; AUC, area under the receiver operating characteristic curve.
