# Supplementary material for: AI-driven high-risk pregnancy prediction: balancing early detection, anxiety, and discrimination in digital public health
Source: Front Public Health. 2026 Mar 26;14:1752484. doi: 10.3389/fpubh.2026.1752484 (PMC13062171; doi:10.3389/fpubh.2026.1752484)
Supplement: Supplementary file 3 [file Table_3.DOCX]

**Table S3. PPH prediction pipeline (full matrix)**

| **Domain/Stage** | **Benefits** | **Harms (anxiety/discrimination)** | **Mitigations** |
| --- | --- | --- | --- |
| **1. Data capture (parity, previa/accreta, labs, uterine tone, EBL)** | Early identification of hemorrhage-prone deliveries; improves readiness | Under-documentation of blood loss or risk factors in low-resource wards biases model | Standardize PPH data capture; validate EBL recording |
| **2. Feature selection & temporal modeling** | Detects evolving hemorrhage risk during labor/CS | Using site-level variables may encode facility inequity | Use patient-level features; separate facility effects |
| **3. Model training** | Predicts need for massive transfusion/uterotonics | FP labeling may drive unnecessary anxiety and elective CS pressure | Balance FP cost; monitor elective CS increase |
| **4. External validation** | Confirms performance across vaginal/CS and referral levels | Under-prediction in rural settings may delay rescue | Validate by delivery mode and setting; subgroup analysis |
| **5. Calibration & thresholds** | Converts scores to actionable readiness tiers | Too sensitive thresholds → “hemorrhage-expectation anxiety” | Local calibration; set tiered thresholds linked to bundles |
| **6. In-room DSS deployment** | Triggers PPH bundles (uterotonics ready, blood products, team alert) | Alarm burden; defensive prophylaxis in low-risk women | Clinician-in-loop; bundle-linked alerts only |
| **7. Communication to patient/family** | Explains need for preparedness (blood, consent) | Fear of maternal death; trauma; blame | Clear, brief absolute-risk communication; reassurance about readiness |
| **8. Post-delivery monitoring** | Earlier detection of secondary PPH | Over-monitoring may reduce comfort, breastfeeding confidence | Tailor monitoring to risk; explain purpose |
| **9. Post-deployment audit** | Tracks PPH outcomes and response time | Drift may cause over/under-use of transfusion | Continuous recalibration; equity-stratified PPH morbidity tracking |

**Abbreviations：**PPH, postpartum hemorrhage; EBL, estimated blood loss; CS, cesarean section; FP, false positive; DSS, decision support system.
