# Supplementary material for: AI-driven high-risk pregnancy prediction: balancing early detection, anxiety, and discrimination in digital public health
Source: Front Public Health. 2026 Mar 26;14:1752484. doi: 10.3389/fpubh.2026.1752484 (PMC13062171; doi:10.3389/fpubh.2026.1752484)
Supplement: Supplementary file 4 [file Table_4.DOCX]

**Table S4. Preterm birth prediction pipeline (full matrix)**

| **Domain/Stage** | **Benefits** | **Harms (anxiety/discrimination)** | **Mitigations** |
| --- | --- | --- | --- |
| **1. Data capture (hx PTB, cervix length, infection markers, stress, sociodemographic)** | Early detection of PTB risk; allows timely progesterone/cerclage/transfer | Social variables may become discriminatory proxies; missing cervix data in low-resource sites | Separate social risk from biologic prediction; document missingness |
| **2. Feature engineering (trajectories, NLP notes)** | Improves prediction using longitudinal patterns | NLP on notes may encode clinician bias | Bias review of text features; fairness checks |
| **3. Training** | Captures heterogeneity (spontaneous vs iatrogenic PTB) | FP increases “waiting anxiety,” over-restriction of activity | Cost-sensitive training; FP monitoring |
| **4. External validation** | Ensures generalizability across GA windows | Under-performance in certain subgroups widens PTB gaps | Multi-population validation; subgroup calibration |
| **5. Calibration & action thresholds** | Aligns risk tiers to interventions/transfer | Over-low thresholds → overtreatment, anxiety | Tiered thresholds mapped to guideline actions |
| **6. DSS deployment (ANC/L&D)** | Triggers antenatal steroids, MgSO₄ neuroprotection, transfer | Alert fatigue; early iatrogenic delivery if over-trusted | Clinician confirmation; audit iatrogenic PTB rate |
| **7. Risk communication** | Supports shared decision-making re interventions | Risk seen as certainty; fear for neonatal outcomes | Absolute risk visuals; emphasize uncertainty and modifiable steps |
| **8. Follow-up & home monitoring** | Enables early symptom triage | Digital divide; anxiety from constant monitoring | Offer low-tech channels; avoid raw risk pushes |
| **9. Post-deployment monitoring** | Tracks PTB prevention impact | Drift or policy shifts change baseline risk | Recalibrate periodically; equity-stratified PTB outcomes |

**Abbreviations：**PTB, preterm birth; hx, history; NLP, natural language processing; FP, false positive; GA, gestational age; DSS, decision support system; ANC, antenatal care; L&D, labor and delivery; MgSO₄, magnesium sulfate.
