# Supplementary material for: AI-driven high-risk pregnancy prediction: balancing early detection, anxiety, and discrimination in digital public health
Source: Front Public Health. 2026 Mar 26;14:1752484. doi: 10.3389/fpubh.2026.1752484 (PMC13062171; doi:10.3389/fpubh.2026.1752484)
Supplement: Supplementary file 5 [file Table_5.DOCX]

**Table S5. Obstetric ICU high-risk prediction pipeline (full matrix)**

| **Domain/Stage** | **Benefits** | **Harms (anxiety/discrimination)** | **Mitigations** |
| --- | --- | --- | --- |
| **1. Cohort definition (ward→HDU→ICU)** | Builds early-warning models for deterioration and ICU need | Referral bias inflates risk; excludes low-access patients | Multi-level cohorts; transparent transfer criteria |
| **2. Feature selection (vitals trends, labs, SOFA/APACHE, hemorrhage/sepsis signals)** | Detects evolving shock/resp failure/MOF earlier | Proxies (insurance, ward type) encode inequity | Clinically grounded features; proxy review |
| **3. Training on rare outcomes** | Improves sensitivity for catastrophic events | FP leads to unnecessary ICU transfer & distress | Rare-event methods; explicit FP cost tuning |
| **4. External validation** | Confirms performance in different hospitals/regions | Under-prediction in vulnerable groups delays rescue | Validate across care levels; subgroup performance |
| **5. Calibration & triage thresholds** | Enables tiered ICU referral and resource planning | Miscalibration → panic, stigma, or missed rescue | Local calibration; tiered thresholds linked to ICU bundles |
| **6. Triage deployment (ED/L&D/wards)** | Real-time alerts to mobilize ICU/rapid response teams | Alert fatigue; defensive over-triage; clinician over-trust | Clinician-in-loop; audit transfer appropriateness |
| **7. Communication around transfer** | Improves understanding of ICU need and safety plan | Risk interpreted as imminent death; separation from newborn anxiety; financial stress | Absolute-risk framing; reassurance of proactive protection; counseling triggers |
| **8. ICU course dynamic prediction** | Guides escalation/de-escalation (ventilation, vasopressors, massive transfusion) | Score-driven over-intervention; patient stress if exposed | Limit patient exposure to raw scores; action-bundle thresholds |
| **9. Governance & post-deployment monitoring** | Supports system QI and maternal safety dashboards | Drift/bias silently increases harms | Continuous recalibration; equity+harm dashboards; transparent update policy |

**Abbreviations：**ICU, Intensive Care Unit; HDU, High-Dependency Unit; SOFA, Sequential Organ Failure Assessment; APACHE, Acute Physiology and Chronic Health Evaluation; MOF, Multiple Organ Failure; FP, False Positive; ED, Emergency Department; L&D, Labor and Delivery; QI, Quality Improvement; resp, respiratory.
